# Karl Landsteiner (1868–1943)

**Published:** 2018-06

**Authors:** Dariush D. Farhud

**Affiliations:** 1. School of Public Health, Tehran University of Medical Sciences, Tehran, Iran; 2. Dept. of Basic Sciences/Ethics, Iranian Academy of Medical Sciences, Tehran, Iran

**Figure:**
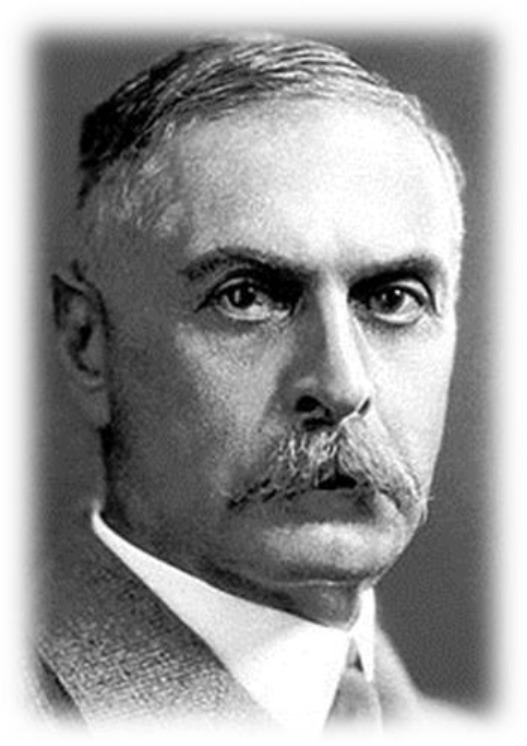
Karl Landsteiner

Karl Landsteiner was born 150 years ago in Vienna on June 14^th^, 1868. He lost his father, Leopold Landsteiner, when he was just 6 years old. His father was a doctor of law and a well-known journalist and newspaper publisher. He was the first editor of Disperse journal in Vienna. After his father died, his mother took his custody. When he finished his school, he studied medicine at Vienna University and graduated in 1891 at the age of 23. He published a paper on influence of diet on the composition of blood ash in 1891. To gain further knowledge of chemistry, he worked in a laboratory with Arthur Hanztch in Zurich, Emil Fischer in Wurzburg and Evigen Bamberger in Munich, for five years. Landsteiner returned to Vienna and resumed his medical studies at a general hospital. He became Max Von Gruber’s assistant in Hygiene Institute in Vienna. At that time, he was interested in immune mechanism and nature of antibodies.

From 1898 till 1908 he was assistant in pathological anatomy at Vienna University. He worked on morbid physiology. In 1901–1903, Lansteiner pointed that same reaction, if transfer the blood from animals to human, blood clot will form and block the vessels, which Landois had reported before, may occur when the blood of one human individual is transfused to other human individuals and that would be the cause of shock, jaundice, and hemoglobinuria. He showed that in combination of different blood groups, the red blood cells that carries oxygen, become slow and have lethal effect. Landsteiner classified the blood in four groups. In 1901, he published a paper about discovery of ABO blood groups (1) (Table 1). Before this, Landsteiner had claimed that blood groups are inherited (2). Because of his valuable discovery, he won the Nobel Prize in medicine, in 1930.

**Table 1:** Major blood groups, year of report, discoverer/s. All references in (1)

***Blood Group***	***Year***	***Reporter (s)***
ABO – System	1901	Landsteiner K
M/N – System	1927	Landsteiner K, Levine P
P – System	1927	Landsteiner K, Levine P
Secretor/Non –(ss)	1932	Schiff F, Sasaki H
Factor Q	1935	Imamuras S
Rhesus (Rh)	1940/41	Landsteiner K, Wiener A
Lutheran (Lu)	1945	Callenders S, Race RR, Paykoc Z
Lewis (Le)	1946	Mourant AE
Kell (K)	1946	Coombs RRA, Mourant AE, Race RR
Factor S/s	1947	Walsh RJ, Montgomery C
Duffy (FY)	1950	Cutbush M, Mollison PL
Kidd (Jk)	1951	Race RR et al.
Diago (Di)	1954	Levine P et al.
Yt System	1956	Eaton BR et al.
Auberger (AU)	1961	Salmon C et al.
Xg	1962	Mann JD et al.
Dombrock (Do)	1965	Swanson J et al.

In 1911 he became professor of pathological anatomy in Vienna University, without the corresponding salary. In 1916 he got his professorial degree at Vienna University and got married with Helen Wlasto. Up to 1919, Landsteiner had published many papers on his findings in morbid anatomy, immunology, serology, virology, histology and bacteriology with a number of collaborators. Meanwhile he discovered new facts about syphilis immunology. In 1919 he went to the Hague in Netherlands and work at a catholic hospital until 1922. In 1922 he migrated to New York with his family and worked at Rockefeller Institute with P. Levine and Alexander Wiener.

In 1939, he retired and became emeritus professor at Rockefeller Institute. He continued his work on blood groups with Wiener and his colleague which had led to the discovery of Rh factor in 1940 (3, 4).

Landsteiner spent his whole life on examining blood groups, antigens, antibodies, and other immunologic agents in the blood.

Karl Landsteiner had a heart attack in his laboratory on June 24^th^, 1943 and died two days later on June 26^th^, 1943 at the age of 75 in the same hospital which he had done such distinguished work.

## References

[1] Farhud DD Zarif Yeganeh M (2013). A brief history of human blood groups. Iran J Public Health, 42(1):1–6. PMC3595629 23514954

[2] Landsteiner K (1901). Ueber Agglutinationsersche inungen normalen menschlichen Blutes. Wien. Klin. Wschr 14, 1132.

[3] Landsteiner K Wiener AS (1940). An agglutinable factor in human blood recognized by Immune sera for rhesus blood. Proc Soc Exp Biol, (N.Y) 43:223.

[4] Landsteiner K Wiener AS (1941). Studies on an agglutinogen (Rh) in human blood reacting with anti-rhesus sera and with human isoantibodies. J Exp Med, 74(4): 309–320. 19871137 10.1084/jem.74.4.309 PMC2135190

